# Serum Cholesterol and Nigrostriatal R2* Values in Parkinson's Disease

**DOI:** 10.1371/journal.pone.0035397

**Published:** 2012-04-17

**Authors:** Guangwei Du, Mechelle M. Lewis, Michele L. Shaffer, Honglei Chen, Qing X. Yang, Richard B. Mailman, Xuemei Huang

**Affiliations:** 1 Department of Neurology, Pennsylvania State University-Milton S. Hershey Medical Center, Hershey, Pennsylvania, United States of America; 2 Department of Pharmacology, Pennsylvania State University-Milton S. Hershey Medical Center, Hershey, Pennsylvania, United States of America; 3 Department of Radiology, Pennsylvania State University-Milton S. Hershey Medical Center, Hershey, Pennsylvania, United States of America; 4 Department of Neurosurgery, Pennsylvania State University-Milton S. Hershey Medical Center, Hershey, Pennsylvania, United States of America; 5 Department of Kinesiology, Pennsylvania State University-Milton S. Hershey Medical Center, Hershey, Pennsylvania, United States of America; 6 Department of Public Health Sciences, Pennsylvania State University-Milton S. Hershey Medical Center, Hershey, Pennsylvania, United States of America; 7 Epidemiology Branch, National Institute of Environmental Health Sciences, Research Triangle Park, North Carolina, United States of America; University of Cambridge, United Kingdom

## Abstract

**Background:**

The occurrence of Parkinson's disease (PD) is known to be associated both with increased nigrostriatal iron content and with low serum cholesterol and PD, but there has been no study to determine a potential relationship between these two factors.

**Methods:**

High-resolution MRI (T1-, T2, and multiple echo T2*-weighted imaging) and fasting lipid levels were obtained from 40 patients with PD and 29 healthy controls. Iron content was estimated from mean R2* values (R2* = 1/T2*) calculated for each nigrostriatal structure including substantia nigra, caudate, putamen, and globus pallidus. This was correlated with serum cholesterol levels after controlling for age, gender, and statin use.

**Results:**

In patients with PD**,** higher serum cholesterol levels were associated with lower iron content in the substantia nigra (R = −0.43, p = 0.011 for total-cholesterol, R = −0.31, p = 0.080 for low-density lipoprotein) and globus pallidus (R = −0.38, p = 0.028 for total-cholesterol, R = −0.27, p = 0.127 for low-density lipoprotein), but only a trend toward significant association of higher total-cholesterol with lower iron content in the striatum (R = −0.34, p = 0.052 for caudate; R = **−**0.32, p = 0.061 for putamen). After adjusting for clinical measures, the cholesterol-iron relationships held or became even stronger in the substantia nigra and globus pallidus, but weaker in the caudate and putamen. There was no significant association between serum cholesterol levels and nigrostriatal iron content for controls.

**Conclusions:**

The data show that higher serum total-cholesterol concentration is associated with lower iron content in substantia nigra and globus pallidus in Parkinson's disease patients. Further studies should investigate whether this is mechanistic or epiphenomenological relationship.

## Introduction

Although the etiology is known in a small percentage of genetically-linked cases, Parkinson's disease (PD) is largely idiopathic, and likely involves interaction of host susceptibility (determined by our genome and/or behavior) and environmental factors. Besides the well documented loss of dopamine neurons in the substantia nigra (SN) [1], increased iron content in the SN also has been found postmortem in PD [2]–[4]. Ferritin-bound iron is paramagnetic, and causes a strong reduction in the proton transverse relaxation time (T2*) [5]. Several brain MRI studies have shown that the proton transverse relaxation rate (R2* = 1/T2*) is correlated with iron concentration in brain tissue [6], [7], and is increased in SN of PD patients [6], [8]–[12]. The exact role of this iron accumulation in PD is, however, unclear.

It is known that genetic alterations of some proteins involved in the regulation of iron homeostasis can cause brain iron accumulation and neurodegenerative disorders in humans [13], [14]. These disorders, grouped under the name “neurodegeneration with brain iron accumulation”, [15] include pathothenate kinase-associated neurodegeneration (PKAN) [16], hereditary aceruloplasminemia [17], and neuroferritinopathy [18]. In addition, iron regulatory protein-2 null mice that have alterations in brain iron homeostasis show neurodegeneration and develop movement abnormalities [19]. These observations suggest the hypothesis that iron dysregulation may have an etiological role in neurodegenerative diseases.

Excessive accumulation of iron in neurons causes toxicity presumed to result from alterations in the ratio of ferric to ferrous iron and subsequent production of toxic hydroxyl radicals [20], [21]. Furthermore, iron deposition in the brain can promote conformational changes in α-synuclein, resulting in aggregation and possible contribution to the pathogenesis of PD [22]. Thus, it may be that iron deposition contributes to brain damage in patients with PD [20], [23], [24]. It also is known that iron binding capacity may be increased with higher metabolism [25]. Furthermore, catecholamine synthesis and degradation requires iron as a co-factor [26] and thus the increased iron in the SN may reflect this increased synthesis. Thus, it also is possible that increased iron content may represent a compensatory change due to primary pathology. Little is known, however, about the factors associated with increased iron accumulation in the SN of PD patients.

Recent studies also suggest higher serum cholesterol may be related to a lower occurrence [27]–[32] and slower progression [33] of PD, although one prospective study offered a contradictory finding [34]. The weight of the evidence clearly favors an inverse association between serum cholesterol and occurrence of PD. Whether serum cholesterol and iron deposition are related in PD, however, is unknown. One study found lower dietary cholesterol intake, particularly with higher iron intake, correlated with an increased risk for PD, suggesting cholesterol and iron may be part of a chain of events leading to the death of dopaminergic neurons [35]. For these reasons, we tested the hypothesis that higher serum cholesterol levels are associated with lower iron accumulation in the SN and related down stream nigrostriatal structures in PD.

## Methods

### Subjects

Forty PD subjects and 29 controls were recruited from patients, their spouses, and relatives presenting to a tertiary movement disorders clinic (see Table 1 for detailed demographic information). PD diagnosis was confirmed by a movement disorder specialist (XH) according to published criteria [36]. Unified PD Rating Scale part III (UPDRS-III) motor scores were obtained for each PD subject after withholding all PD medication overnight (∼12 h). The levodopa equivalent dose (LEDD) was estimated for PD subjects according to a published formula [37]. All subjects gave written informed consent that was consistent with the Declaration of Helsinki, and this study was reviewed and approved by the Penn State Hershey Institutional Review Board.

**Table 1:** Overall characteristics of study subjects.

	Parkinson's disease	Controls	p-value
***Demographics***			
No. (female, male)	40 (17, 23)	29 (17, 12)	0.227^a^
Age, years (mean±SD, y)	60.7±8.3	59.6±6.7	0.551^b^
UPDRS-III motor scores (mean±SD)	23.4±15.2	-	N/A
Disease duration, years (mean±SD, y)^d^	4.2±4.7	-	N/A
Hoehn and Yahr Stage (I/II/III)	14/22/4	-	N/A
Levodopa equivalent dose (LEDD, mg)	532±395	-	N/A
Statin use (yes/no)	9/31	6/23	1.000
***Serum fasting cholesterol levels***			
Total-cholesterol (mean±SD, mg/dL)	195±36	201±39	0.852^c^
LDL-cholesterol (mean±SD, mg/dL)	123±29	121±34	0.607^c^
R2* values in nigrostriatal pathways			
SN	34.9±5.7	30.4±4.6	0.001^c^
Caudate	22.3±3.5	22.4±2.6	0.720^c^
Putamen	28.4±4.7	27.3±3.1	0.394^c^
GP	37.2±6.0	35.6±4.2	0.117^c^

a p-value is calculated from Fisher's exact test.

b p-value is calculated from two-sample Student's t-test with equivalent variance.

c p-value is calculated from ANCOVA with adjustment for age, gender, and use of statins.

d Disease duration is defined as the years since diagnosis.

### Determination of fasting serum total and low density lipoprotein (LDL)-cholesterol levels

Four milliliter blood specimens were obtained from all subjects after overnight fasting. The blood samples were allowed to clot for 30 min and then centrifuged within one hour after collection until clot and serum were clearly separated. The serum then was transferred into a plastic vial and serum lipids were measured by standard biochemical methods as described in the Vitros Chemistry product manual (Ortho-Clinical Diagnostics, Inc., Rochester, NY) at the Penn State Milton S. Hershey Medical Center Clinical Laboratory.

### MRI Data acquisition

All subjects were scanned using a 3.0 tesla MR Scanner (Trio, Siemens Magnetom, Erlangen, Germany, with an 8-channel phased array head coil) and high-resolution T1- and T2-weighted images, along with T2*-weighted (multiple gradient echoes) images, were collected. A magnetization-prepared rapid acquisition gradient echo sequence was used to obtain T1-weighted images with TR = 1540 ms, TE = 2.34 ms, field of view = 256 mm, matrix = 256×256, slice thickness = 1 mm (with no gap), slice number = 176. T2-weighted images were collected using a fast-spin-echo sequence with TR = 2500, TE = 316, with the same resolution configuration as that for T1-weighted images. A multiple gradient echoes sequence was used to estimate the proton transverse relaxation rate, R2* (R2* = 1/T2*) [38]. Six echoes with TEs ranging from 7 to 47 ms and an interval of 8 ms were acquired with TR = 54 ms, flip angle = 20°, field of view = 256 mm, matrix = 256×256, slice thickness = 1 mm (with no gap), slice number = 64.

### Image processing and analysis

#### Segmentation of regions of interest (ROIs)

**Figure 1:**
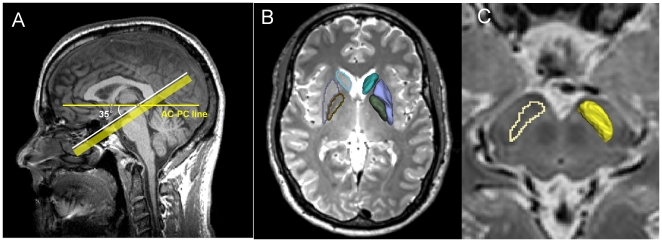
The segmentation procedure for the substantia nigra and nigrostriatal structures. An illustration of reformatting position used for the segmentation of the substantia nigra (1A) and the segmentation example of the nigrostriatal structures (1B and 1C).

The SN was delineated manually. Individual high resolution T2-weighted images were reformatted by positioning the AC-PC line to the center of the image and then rotating the AC-PC line 35° superior to the PC point and along the mid-sagittal plane, as shown in Figure 1A. The 35° rotation was used to minimize the inclusion of the subthalamic nucleus that lays superior-anterolateral to the SN [39], [40]. The SN was defined as a hypo-intensity band between the red nucleus and cerebral peduncle in axial sections on the multiplanar reformatted T2-weighted images. The segmentation of the SN was started at one slice lower than the level of the red nucleus showing the largest radius. A total of five slices (from the dorsal to ventral parts of the SN, 5 mm height) were used as the ROI of the SN. The level of the SN is shown as the yellow band in Figure 1A. ITK-SNAP (www.itksnap.org) was used to perform manual segmentation, whereas an in-house multi-plane reformation tool was used to reformat T2-weighted images and then transform the ROIs from the reformatted coordinate space to the original coordinate space for further analysis [41]. The reasons the SN is treated as one ROI instead of separating it into the pars compacta and pars reticulata subregions are 1) the exact demarcation of the pars compacta and pars reticulata on T2-weighted images still remains controversial according to recent studies [6], [42]–[45]; and 2) the distribution of dopaminergic neurons within the substantia nigra is quite heterogeneous and possibly spreads into the pars reticulata. [46], [47]. The putamen, caudate, and global pallidus (GP) were segmented by using a probabilistic atlas-based automatic segmentation software AutoSeg (Neuro Image Research and Analysis Laboratories, University of North Carolina at Chapel Hill, NC, USA) on T1-weighted and T2-weighted images [48], [49]. The segmentation results are illustrated in Figures 1B and 1C.

#### R2* maps

The multiple gradient echoes images were used to estimate the R2* maps by using a voxel-wise linear least-squares fit to a mono-exponential function with free baseline using an in-house MATLAB (The MathWorks, Inc., Natick, MA) tool. An affine registration process implemented in 3D Slicer (www.slicer.org) then was used to map ROIs to the R2* maps and reduce motions artifacts between T2-weighted and multiple gradient echoes T2* images by co-registering T2-weighted images to an averaged magnitude image of the six echoes. For each individual subject, the mean R2* values of the SN were calculated by using the trimmed mean (from the 5%–95% percentile) of all voxels in the ROI to reduce variability introduced by potential segmentation and/or registration errors. We used trimmed means because they represent a more robust central estimator of the measurement, and they are based on the assumption that the top and bottom 5% values represent potential errors.

### Statistical Analysis

The demographic data of PD subjects and controls were compared using two-sample t-tests and Fisher's exact tests as appropriate. Regional R2^*^ values and serum cholesterol levels were compared separately between PD and controls by analyses of covariance with adjustments for age, gender, and the use of cholesterol lowering drugs (statins). The correlations between R2* values and serum cholesterol levels in PD and control subjects were determined using Spearman's partial correlation coefficients (robust to departures from normality, including the presence of outliers), with adjustment for the effects of age, gender, and statin use in both PD and controls separately. The relationship between UPDRS-III scores, disease duration, LEDD, and both R2* values and serum cholesterol levels were explored by Spearman's partial correlation analysis in the PD group. The statistics are presented without a multi-comparison correction because the explored variables are intrinsically dependent and the exploratory nature of this study. All statistical analyses were performed using SAS 9.2 (SAS Institute Inc., Cary, NC, USA).

## Results

As shown in Table 1, there were no significant differences between PD and controls in terms of age, gender, or statin use status. R2* values in the SN of PD subjects were significantly higher compared to that of controls. There were no significant differences between PD and controls in either total-cholesterol or LDL-cholesterol measurements. As shown in Table 2 (and Figure S2), the R2* values in the SN were associated with all three clinical measurements [i.e., UPDRS-III, disease duration, and LEDD]. UPDRS-III motor scores were negatively correlated with serum cholesterol levels in PD subjects, but only reached statistical significance with LDL-cholesterol levels (R = −0.397, p = 0.020) and not total-cholesterol (R = −0.334, p = 0.054). Neither of the serum cholesterol levels was correlated with the other two clinical measurements.

**Table 2:** Spearman's partial correlation coefficients and p-value (in parentheses) between clinical scores and R2* values or serum cholesterol levels.

		Clinical measurements		
		UPDRS-III motor scores	Disease Duration	LEDD
Lipid profiles	Total-cholesterol	−0.334 (0.054)	−0.175 (0.322)	−0.052 (0.768)
	LDL-cholesterol	***−0.397 (0.020)***	−0.062 (0.727)	0.040 (0.824)
R2* in brain regions	SN	***0.355 (0.039)***	***0.418 (0.014)***	***0.407 (0.017)***
	Caudate	***0.397 (0.020)***	0.055 (0.758)	0.266 (0.128)
	Putamen	***0.431 (0.011)***	0.144 (0.417)	***0.424 (0.012)***
	GP	−0.142 (0.424)	−0.061 (0.732)	0.130 (0.464)

*Bold-italicized numbers are statistically significant results with p<0.05 (p value in parentheses).*

*UPDRS-III represents Unified Parkinson's Disease Rating Scale-Motor part;*

*LEDD represents levodopa equivalent dosage.*

When regression analyses between serum cholesterol and iron measurements were performed, there were two outliers (one in each group) detected with regard to serum cholesterol levels (shown in Figure S1). Each of these subjects had extremely high cholesterol levels despite the fact that both subjects were taking cholesterol-lowering drugs. The serum cholesterol level was 290 mg/dL (196 mg/dL LDL-cholesterol) for the PD subject and 285 mg/dL (204 mg/dL LDL-cholesterol) for the control, with all values greater than two times the standard deviation of the overall groups. Because these extreme outliers may bias the correlations analysis, the remaining results come from 39 PD subjects and 28 controls with the two outliers excluded. The correlation analysis results (including those two outliers) are presented in the supplementary material (see Table S1 and Figure S1).

**Figure 2:**
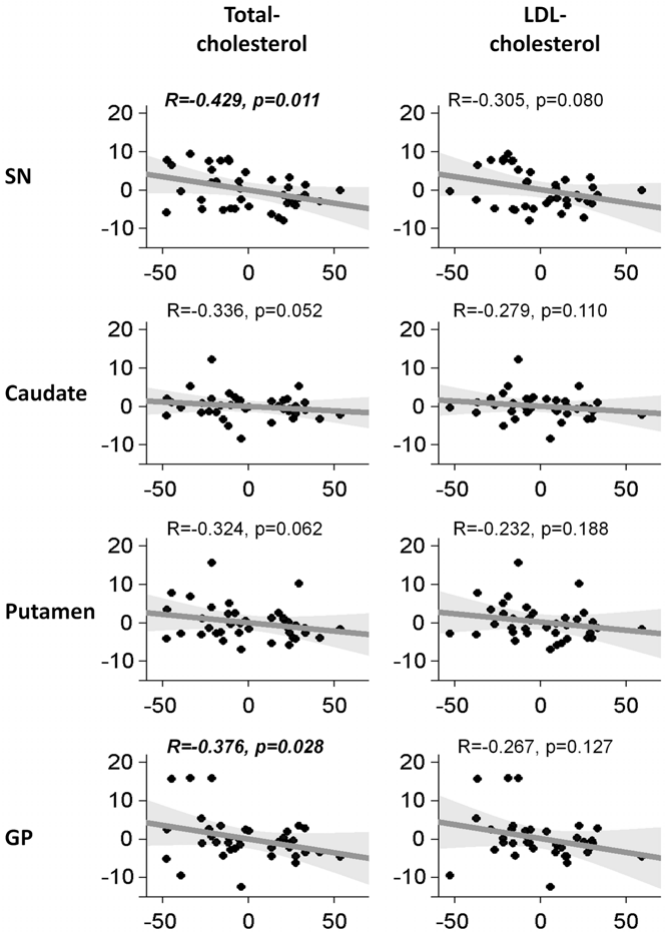
Correlation between nigrostriatal iron and serum cholesterol levels. Partial residual plots of nigrostriatal iron deposition and serum cholesterol levels in Parkinson's disease subjects. X-axes are serum cholesterol levels; y-axes are R2* values controlling for age, gender, and statin use. R and p-values are from Spearman's partial correlation analysis.

As seen in Table 3 and Figure 2, after controlling for age, gender, and statin use, higher serum cholesterol levels were associated with lower iron content in the SN of PD subjects. The association reached statistical significance with total-cholesterol level (R = −0.43, p = 0.011), but not for LDL-cholesterol level (R = −0.30, p = 0.080). Higher serum cholesterol levels also were correlated with lower iron content in the GP in PD subjects. The association reached statistical significance with total-cholesterol level (R = −0.38, p = 0.028), but not with LDL-cholesterol level (R = −0.27, p = 0.127). The relationship between total-cholesterol and iron content in the SN of PD subjects remained after adjusting either individually (data not shown) or collectively for the three clinical scores (UPDRS-III motor scores, duration of illness, and LEDD). The association between both cholesterol levels and iron content in the GP was even stronger after adjusting for clinical scores (see Table 3 and Figure S2).

**Table 3:** Spearman's partial correlation coefficients and p-values (in parentheses) between regional R2* and serum cholesterol levels in PD and Controls.

	Controls		Parkinson's disease			
	*(adjusted for age, gender, and statin use)*		*(adjusted for age, gender, and statin use)*		*(adjusted for age, gender , statin use, and clinical scores* * *)*	
	Total-cholesterol	LDL-cholesterol	Total-cholesterol	LDL-cholesterol	Total-Cholesterol	LDL-cholesterol
SN	0.163 (0.458)	0.213 (0.329)	***−0.429 (0.011)***	−0.305 (0.080)	***−0.391 (0.030)***	−0.287 (0.118)
Caudate	−0.339 (0.114)	−0.226 (0.299)	−0.336 (0.052)	−0.279 (0.110)	−0.307 (0.093)	−0.209 (0.260)
Putamen	−0.360 (0.091)	−0.243 (0.264)	−0.324 (0.062)	−0.232 (0.188)	−0.329 (0.071)	−0.199 (0.283)
GP	−0.063 (0.775)	−0.116 (0.599)	***−0.376 (0.028)***	−0.267 (0.127)	***−0.552 (0.001)***	***−0.477 (0.007)***

*Bold-italicized numbers are statistically significant results with p<0.05 (p value in parentheses).*

* *Clinical scores include UPDRS-III motor scores, disease duration, and LEDD.*

There was a trend for an association between total-cholesterol and iron content in the striatum (R = −0.34, p = 0.052 for caudate; R = **−**0.32, p = 0.062 for putamen) in the PD group, but the trend was weakened after adjusting for clinical scores. The association between LDL-cholesterol level and iron content in PD subjects was not significant in striatal structures, and similar to total-cholesterol, the relationship was further weakened after adjusting for clinical scores (see Table 3). In controls, serum total-cholesterol was not significantly associated with iron content in the SN (R = 0.16, p = 0.46), striatum (R = −0.34, p = 0.11 for caudate; R = −0.36, p = 0.091 for putamen), or GP (R = −0.063, p = 0.78), nor was LDL-cholesterol level correlated with iron content in any nigrostriatal structure (see Table 3).

## Discussion

The current study was designed to be a first test of the hypothesis that iron contents in SN and serum cholesterol levels were associated in PD. This study provides the first evidence that higher serum cholesterol in PD patients may be associated with lower iron content in the SN and GP. This relationship is not present in age- and gender-matched controls, suggesting that this is a PD- or neurodegeneration-specific association. The data therefore support future efforts to test the hypothesis that cholesterol and iron may be interwoven into a chain of events that relates either to the death of dopamine neurons, and/or compensatory changes in PD. On the other hand, these data cannot rule out the null hypothesis that they are two unrelated changes that occur in PD (see Introduction). The mechanism and implication of the cholesterol-iron association at iron enriched brain structures in PD need to be explored further, and may shed light on understanding the pathoetiology or physiology of PD and related disorders.

Consistent with previous reports [6], [8]–[12], PD subjects demonstrated a significant increase in the R2* value in the SN compared to controls in the present study. In addition, we found a significant correlation between the SN R2* value and all three clinical scores in PD. This is consistent with the idea that R2* in the SN may have utility as an imaging marker for PD progression. Unlike the previous study [6], and considering the exploratory aspect of the present work and controversies surrounding the definition of the subregions of the SN [6], [42], [43], [45], we separated neither the two subregions of the substantia nigra, nor the more- and less-affected sides. Consequently, one limitation of our study is that we could not explore the relationship between R2* and clinical measurements in a more detailed way as was done in these previous studies [6]. The PD group in the present work has a slightly broader spread in the severity of disease than the previous studies [6], [11], [12] that may explain why we were able to find a stronger correlation between R2* values and the clinical measurements. Nevertheless, similar to the previous studies, the PD subjects in the current study are relatively early in their disease course. Additional researches with larger sample sizes that include more advanced stage disease patients are warranted to test this hypothesis more rigorously.

Two previous case-control studies reported significantly lower cholesterol in PD than in controls, each study involving more than 100 PD and 100 controls [28], [50]. The current study of 40 PD and 29 controls is clearly underpowered to detect any significant group difference between PD and control groups for these measurements, and also had gender ratios that were different between PD and control groups. Despite this lack of significant group differences, mean total-cholesterol level, LDL-cholesterol level, and their standard deviations in the current study, however, are very similar to what we reported previously [27], where 124 PD and 112 control subjects were studied. In this previous study [27], higher cholesterol level quartiles were associated with lower prevalence of PD, although the sample size for the current study was too small for a similar analysis. Also like the current study, Huang et al. [27] did not find an association between cholesterol levels and duration of illness. The current data does suggest that in the PD group, higher cholesterol levels seemed to be associated with better UPDRS-III motor scores, consistent with the recent report of Ikeda et al. [50]. It is possible that severe clinical disease is marked by higher iron content in the SN, resulting in changes in cholesterol profiles through malnutrition or behavioral changes. In our study, however, after further adjusting for clinical scores (the UPDRS-III motor scores, disease duration, and LEDD), the relationship between cholesterol levels and iron in both the SN and GP in PD subjects remained (in SN) or became even stronger (in GP). These result are not consistent with the notion that disease-induced motor change or drug use are the primary cause of the cholesterol-iron link in PD, but future studies with parallel assessments of nutritional status are needed to address this question directly.

Although there was no overall difference in iron accumulation in the GP between PD and controls, higher cholesterol in PD was associated with lower iron content in this structure. This association is also PD-specific and not observed in controls. Whereas the GP, like the SN, is known to be a “sink” for metals (e.g., Fe, Mn, Cu) [51]–[54] and is lack in transferrin-binding sites [55], it does not contain dopamine neurons, but it is vulnerable to iron-mediated (e.g., Hallervorden Spatz disease, PKAN and oxidative stress-related neuronal and glial damage such as that observed due to carbon monoxide toxicity [20]. This implies that the cholesterol-iron association in PD is most likely specific to iron-rich and oxidative stress-vulnerable structures, but is not necessarily dopamine neuron-mediated. The exact reasons for this cholesterol-iron correlation in PD are unknown and worth exploration.

The current study has several limitations. First, although our sample size of 40 patients and 29 controls is substantial compared to some other PD neuroimaging studies, it is a relatively small sample size for a cross-sectional study. Second, of those subjects participating in the study, nine PD patients and six controls used statins. Statin use may affect these data in complicated ways because these drugs not only modify cholesterol levels, but also have been hypothesized to have anti-neurodegenerative properties [56], [57]. In the current study, the potential effects caused by statins were adjusted by using partial correlation analysis with statin use as a covariate. We did not exclude subjects taking statins from the analyses due to sample size concerns and also because such exclusion would have differentially excluded subjects with higher cholesterol levels. We did, however, obtain the historical, pre-statin fasting total- and LDL-cholesterol levels from the medical records of statin users. The association between cholesterol levels and iron content in nigrostriatal structures was essentially unaffected by this (data not shown). Although the current data are consistent with the hypothesis that lower cholesterol may contribute to brain iron accumulation and later neurodegeneration, other possibilities exist that should be addressed in future studies.

In summary, this study is the first relating serum cholesterol levels and iron content in nigrostriatal structures in PD. Future studies, preferably prospectively following a large group of PD patients with a statistical analysis strategy that corrects for multiple comparison and a, feasible strategy for separating the SN into the pars compacta and pars reticulata, clearly are warranted, and may shed light on understanding the pathoetiology and/or physiology of PD and related disorders.

## Supporting Information

Figure S1 **Partial residual plots of nigrostriatal iron deposition and serum total-cholesterol levels with the outliers.** X-axes are serum total-cholesterol level; y-axes are R2* values controlling for age, gender, and statin use. R and p-values are from Spearman's partial correlation analysis. The two outliers are detected on the positive side of the x dimension in each group.
(DOC)

Figure S2 Scatter-plots of clinical measures with both R2* and serum cholesterol levels. LEDD = levodopa-equivalent daily dosage.
(DOC)

Table S1 Spearman's partial correlation coefficients and p-values (in parentheses) between R2* and serum cholesterol levels in Parkinson's disease and control groups after controlling for age, gender, and statin use (includes outliers).
(DOC)
