# Supplementary figures and images for: Serum Cholesterol and Nigrostriatal R2* Values in Parkinson's Disease
Source: PLoS One. 2012 Apr 17;7(4):e35397. doi: 10.1371/journal.pone.0035397 (PMC3328461; doi:10.1371/journal.pone.0035397)

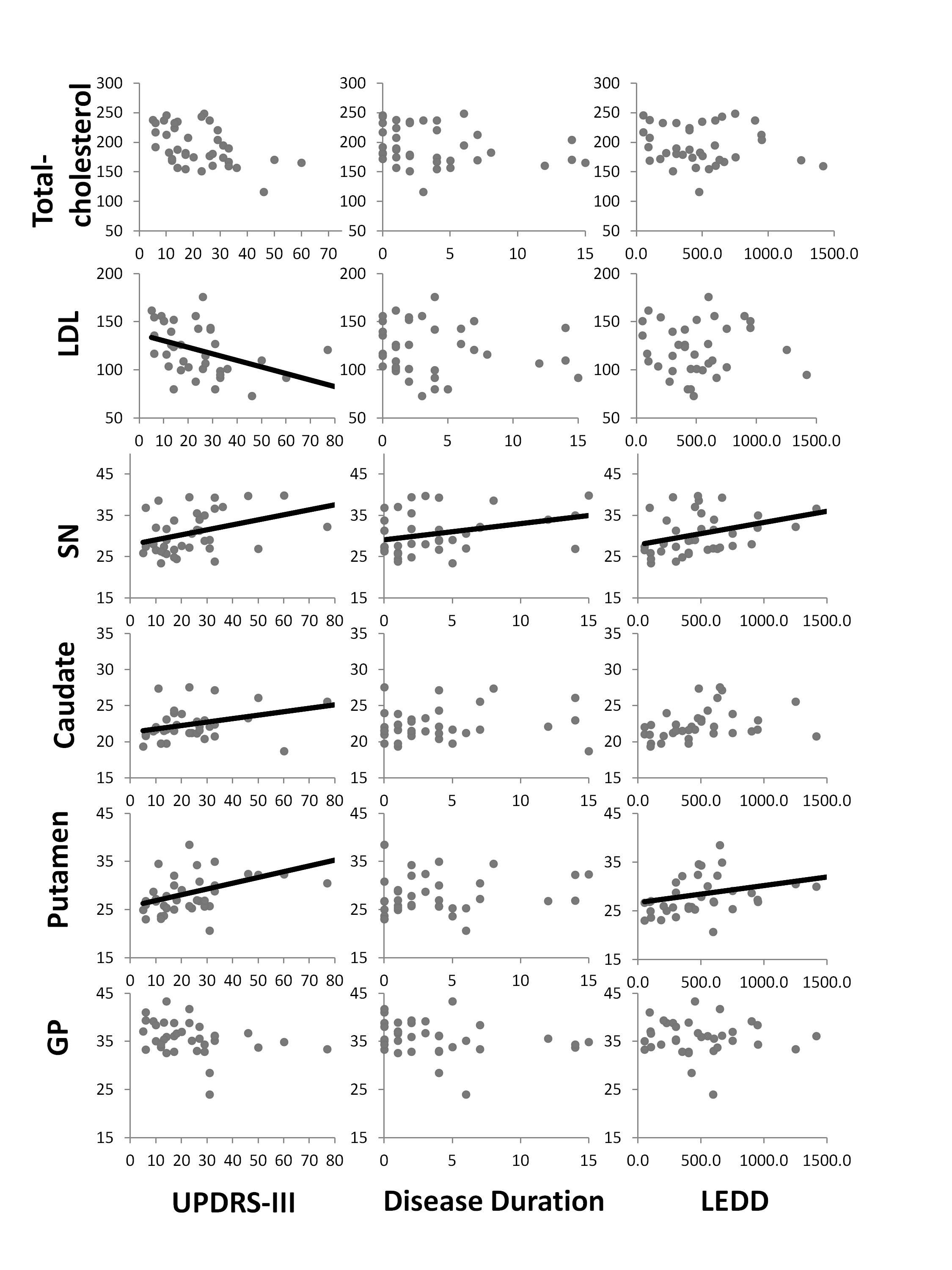

Supplement: Figure S2 — Scatter-plots of clinical measures with both R2* and serum cholesterol levels. LEDD = levodopa-equivalent daily dosage. (DOC) [file pone.0035397.s002.doc]
