# Supplementary material for: Serum Cholesterol and Nigrostriatal R2* Values in Parkinson's Disease
Source: PLoS One. 2012 Apr 17;7(4):e35397. doi: 10.1371/journal.pone.0035397 (PMC3328461; doi:10.1371/journal.pone.0035397)
Supplement: Table S1 — Spearman's partial correlation coefficients and p-values (in parentheses) between R2* and serum cholesterol levels in Parkinson's disease and control groups after controlling for age, gender, and statin use (includes outliers). (DOC) [file pone.0035397.s003.doc]

|  | PD | | Controls | |
| --- | --- | --- | --- | --- |
|  | Total-cholesterol | LDL-cholesterol | Total-cholesterol | LDL-cholesterol |
| SN | ***-0.337 (0.048)*** | -0.2496 (0.1481) | 0.0963 (0.654) | 0.1594 (0.457) |
| Caudate | ***-0.407 (0.017)*** | ***-0.3402 (0.046)*** | ***-0.4387 (0.032)*** | -0.3371 (0.107) |
| Putamen | ***-0.366 (0.031)*** | -0.2750 (0.110) | ***-0.4511 (0.027)*** | -0.3439 (0.100) |
| GP | -0.2504 (0.147) | -0.1763 (0.311) | -0.1911 (0.371) | -0.2071 (0.331) |
