# Supplementary material for: Serum Cholesterol and Nigrostriatal R2* Values in Parkinson's Disease
Source: PLoS One. 2012 Apr 17;7(4):e35397. doi: 10.1371/journal.pone.0035397 (PMC3328461; doi:10.1371/journal.pone.0035397)
Supplement: Supplementary file 3 [file pone.0035397.s001.doc]

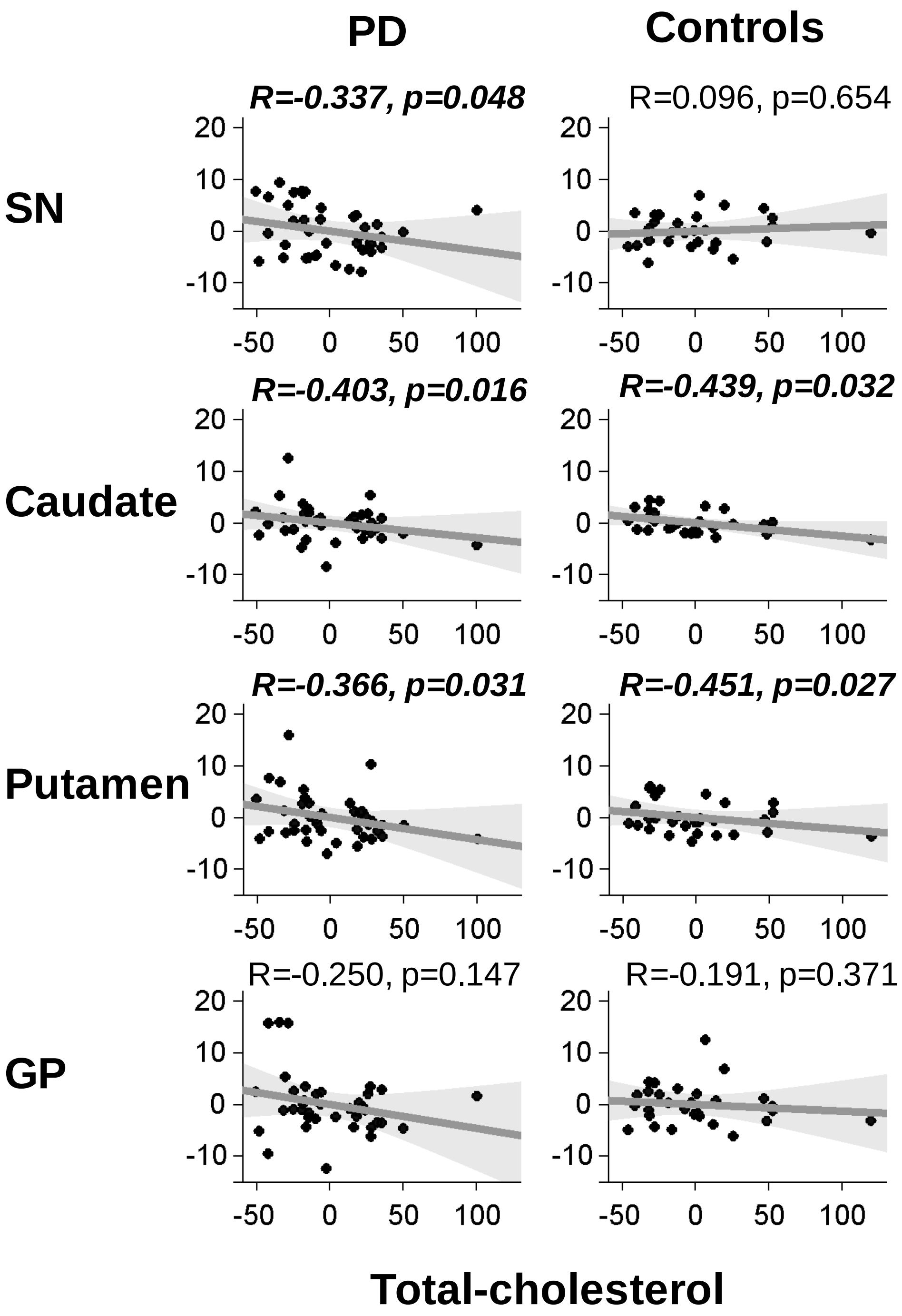

PD
Controls
SN
Caudate
Putamen
GP
R=-0.337, p=0.048
R=0.096, p=0.654
R=-0.403, p=0.016
R=-0.439, p=0.032
R=-0.366, p=0.031
R=-0.451, p=0.027
R=-0.250, p=0.147
R=-0.191, p=0.371
Total-cholesterol
